# Practical applications of deep learning: classifying the most common categories of plain radiographs in a PACS using a neural network

**DOI:** 10.1007/s00330-020-07241-6

**Published:** 2020-09-28

**Authors:** Thomas Dratsch, Michael Korenkov, David Zopfs, Sebastian Brodehl, Bettina Baessler, Daniel Giese, Sebastian Brinkmann, David Maintz, Daniel Pinto dos Santos

**Affiliations:** 1 Institute of Diagnostic and Interventional Radiology, University Hospital Cologne, Kerpener Str. 62, 50937 Cologne, Germany; 2 Institute of Computer Science, Johannes Gutenberg University Mainz, Mainz, Germany; 3 Institute of Diagnostic and Interventional Radiology, University Hospital Zurich, Zürich, Switzerland; 4 Department of General, Visceral and Cancer Surgery, University Hospital Cologne, Cologne, Germany

**Keywords:** Machine learning, Radiography, Artificial intelligence

## Abstract

**Objectives:**

The goal of the present study was to classify the most common types of plain radiographs using a neural network and to validate the network’s performance on internal and external data. Such a network could help improve various radiological workflows.

**Methods:**

All radiographs from the year 2017 (*n* = 71,274) acquired at our institution were retrieved from the PACS. The 30 largest categories (*n* = 58,219, 81.7% of all radiographs performed in 2017) were used to develop and validate a neural network (MobileNet v1.0) using transfer learning. Image categories were extracted from DICOM metadata (study and image description) and mapped to the WHO manual of diagnostic imaging. As an independent, external validation set, we used images from other institutions that had been stored in our PACS (*n* = 5324).

**Results:**

In the internal validation, the overall accuracy of the model was 90.3% (95%CI: 89.2–91.3%), whereas, for the external validation set, the overall accuracy was 94.0% (95%CI: 93.3–94.6%).

**Conclusions:**

Using data from one single institution, we were able to classify the most common categories of radiographs with a neural network. The network showed good generalizability on the external validation set and could be used to automatically organize a PACS, preselect radiographs so that they can be routed to more specialized networks for abnormality detection or help with other parts of the radiological workflow (e.g., automated hanging protocols; check if ordered image and performed image are the same). The final AI algorithm is publicly available for evaluation and extension.

**Key Points:**

*• Data from one single institution can be used to train a neural network for the correct detection of the 30 most common categories of plain radiographs.*

*• The trained model achieved a high accuracy for the majority of categories and showed good generalizability to images from other institutions.*

*• The neural network is made publicly available and can be used to automatically organize a PACS or to preselect radiographs so that they can be routed to more specialized neural networks for abnormality detection.*

## Introduction

Machine learning is predicted to have a huge impact on the field of radiology [1], augmenting and assisting radiologists [2]. With new papers being published every week, one central question remains: What can machine learning do for the average radiologist? Currently, the majority of research in radiology seems to be focused on applying machine learning to the parts of the imaging pipeline that involve perception and reasoning (e.g., detection, quantification, and diagnostic reasoning) [3, 4]. However, due to various barriers (e.g., ethical, economical, and legal), this approach, while promising, may not be the optimal starting point for introducing artificial intelligence into the radiological workflow. Instead, artificial intelligence could be used as a tool for quality assurance and help with automating simple but tedious task encountered in clinical routine [5]. For example, one common challenge in a picture archiving and communication system (PACS) is that images are often labeled incorrectly in the corresponding DICOM tag. The problem of unreliable DICOM information was first demonstrated by Güld et al who found that the DICOM tag *Body Part Examined* was incorrect in 15.3% of cases [6]. This is not only problematic for the retrieval of images for the purpose of creating datasets but also hinders the development of imaging pipelines in which images are automatically routed to specific classification algorithms. Besides, many other parts of the radiological workflow rely on correctly labeled images. Thus, a neural network that can correctly classify and tag images could be used to check that exams are not repeated unnecessarily, control that the acquired image is the same as the one that was ordered, and streamline hanging protocols for optimal reporting on images. Because plain radiographs are still the most common type of imaging performed, a network for the classification of plain radiographs can have a meaningful impact on the radiological workflow. Therefore, the main goal of our study was to develop and validate a convolutional network to classify the most common types of plain radiographs (e.g., thorax pa, abdomen lateral). The final model will be made publicly available so that it can be evaluated and integrated into the radiological workflow.

## Materials and methods

### Radiographs

All radiographs from the year 2017 (*N* = 71,274) performed at our institution were retrieved from the PACS and categorized into 102 categories based on their DICOM metadata (study, series, and image description) according to the WHO manual of diagnostic imaging [7]. Because some categories contained only a small number of images, we limited ourselves to the 30 largest categories (*n* = 58,219), which accounted for 81.7% (58,219/71274) of all radiographs performed in the year 2017 at our institution. For these 30 categories, all images were reviewed again by one radiologist and misclassifications were corrected (i.e., discrepancies between DICOM information and actual image content). Table 1 shows the final dataset with all categories selected for the study and the number of images per class. For each of the 30 categories, 100 randomly selected images were set aside for internal validation (*n* = 3000) and the rest of the images was used as the training set (*n* = 55,219). To assess the generalizability of the results, we used images from other institutions, acquired with machines from multiple vendors, stored in our PACS (*n* = 5324) as an external validation set. To ensure that these images were labeled correctly, these images were manually labeled by two experienced radiologists because DICOM information could not be automatically processed, was missing, or was in several different languages. These images were not part of the training set and only used to validate the trained network. Table 1 shows the number of images per category for the external validation set.

**Table 1:** Images per category used for training the network, internal validation, and external validation

Category	Images from own institution	Training	Internal validation	External validation
Abdomen AP	1743	1643	100	218
Abdomen left lateral decubitus	340	240	100	29
Ankle AP	1236	1136	100	75
Ankle lateral	1200	1100	100	94
Cervical spine AP	1209	1109	100	100
Cervical spine lateral	1330	1230	100	150
Chest lateral	7480	7380	100	981
Chest PA/AP	14,217	14,117	100	1114
Elbow AP	1060	960	100	135
Elbow lateral	1136	1036	100	123
Finger AP	664	564	100	30
Finger lateral	793	693	100	28
Foot AP	1234	1134	100	126
Foot oblique	1130	1030	100	106
Hand AP	1683	1583	100	220
Hand oblique	1525	1425	100	195
Hip joint oblique lateral	1409	1309	100	105
Knee AP	2095	1995	100	142
Knee lateral	2045	1945	100	118
Lumbar spine AP	2414	2314	100	166
Lumbar spine lateral	3398	3298	100	233
Panoramic Radiograph	475	375	100	4
Patella axial	842	742	100	10
Pelvis AP	2022	1922	100	113
Shoulder AP	1048	948	100	166
Shoulder outlet	867	767	100	117
Thoracic spine AP	778	678	100	87
Thoracic spine lateral	858	758	100	100
Wrist AP	973	873	100	116
Wrist lateral	1015	915	100	123
Total	58,219	55,219	3000	5324

### Neural network training

All images were exported from the PACS as JPEG (Joint Photographic Expert Group) images and anonymized in the process. Using the images in the training set (*n* = 58,219), a pretrained MobileNet (Version 1.0) was retrained using oversampling—to account for imbalanced classes—with 22,000 training steps and a learning rate of 0.1. No image augmentation techniques were used. The network was trained on a standard MacBook Pro (Retina, 15–in., Late 2013, 16-GB DDR RAM, 2.3-GHz Quad-Core Intel Core i7).

### Statistical analysis

Performance metrics, such as sensitivity, specificity, positive predictive value, and negative predictive value, were calculated using SPSS Version 26.0 [8].

## Results

### Internal validation

In the internal validation, the overall accuracy of the model in the validation set was 90.3% (95%CI: 89.2–91.3%). Because in this validation set the number of images in each class was equal (*n* = 100), the average sensitivity was the same as the accuracy (90.3%), indicating that, on average, 90.3% of images in each category were correctly classified by the model (see Table 2 for performance metrics for each individual class). As Table 2 shows, the distribution of the sensitivity of the model was rather balanced across categories, ranging between 61.0 and 100.0%. Eighteen out of 30 categories (60.0%) reached a sensitivity of over 90.0%, and 27 out of 30 categories (90.0%) reached a sensitivity of over 80.0%. Only the categories ankle lateral (sensitivity: 79%), lumbar spine lateral (sensitivity: 77%), and shoulder outlet (sensitivity: 61%) reached a sensitivity below 80.0%.

**Table 2:** Performance metrics for the internal validation

Category	Number of images	Sensitivity		Specificity		PPV		NPV	
		Percent	95%CI	Percent	95%CI	Percent	95%CI	Percent	95%CI
Abdomen AP	100	89	82.9–95.1	99.7	99.5–99.9	90.8	85.1–96.5	99.6	99.4–99.8
Abdomen left lateral decubitus	100	100	100.0–100.0	99.9	99.8–100.0	98	95.3–100.0	100	100.0–100.0
Ankle AP	100	80	72.2–87.8	99.4	99.1–99.7	82.5	74.9–90.0	99.3	99.0–99.6
Ankle lateral	100	79	71.0–87.0	100	99.9–100.0	98.8	96.3–100.0	99.3	99.0–99.6
Cervical spine AP	100	96	92.2–99.8	100	99.9–100.0	99	97.0–100.0	99.9	99.7–100.0
Cervical spine lateral	100	97	93.7–100.0	99.8	99.7–100.0	95.1	90.9–99.3	99.9	99.8–100.0
Chest lateral	100	100	100.0–100.0	99.9	99.7–100.0	96.2	92.5–99.8	100	100.0–100.0
Chest PA/AP	100	100	100.0–100.0	99.8	99.6–99.9	93.5	88.8–98.1	100	100.0–100.0
Elbow AP	100	89	82.9–95.1	98.9	98.6–99.3	74.2	66.3–82.0	99.6	99.4–99.8
Elbow lateral	100	96	92.2–99.8	99.6	99.3–99.8	88.1	82.0–94.2	99.9	99.7–100.0
Finger AP	100	82	74.5–89.5	99.4	99.1–99.7	82	74.5–89.5	99.4	99.1–99.7
Finger lateral	100	81	73.3–88.7	99.3	99.1–99.6	81	73.3–88.7	99.3	99.1–99.6
Foot AP	100	92	86.7–97.3	99.6	99.3–99.8	87.6	81.3–93.9	99.7	99.5–99.9
Foot oblique	100	95	90.7–99.3	99.9	99.7–100.0	96	92.1–99.8	99.8	99.7–100.0
Hand AP	100	84	76.8–91.2	99.7	99.5–99.9	91.3	85.5–97.1	99.4	99.2–99.7
Hand oblique	100	89	82.9–95.1	99.8	99.6–100.0	93.7	88.8–98.6	99.6	99.4–99.8
Hip joint oblique lateral	100	91	85.4–96.6	99.9	99.8–100.0	96.8	93.3–100.0	99.7	99.5–99.9
Knee AP	100	98	95.3–100.0	99.9	99.7–100.0	96.1	92.3–99.8	99.9	99.8–100.0
Knee lateral	100	93	88.0–98.0	99.6	99.3–99.8	87.7	81.5–94.0	99.8	99.6–99.9
Lumbar spine AP	100	94	89.3–98.7	99.9	99.8–100.0	97.9	95.1–100.0	99.8	99.6–100.0
Lumbar spine lateral	100	77	68.8–85.2	100	99.9–100.0	98.7	96.2–100.0	99.2	98.9–99.5
Panoramic Radiograph	100	99	97.0–100.0	100	100.0–100.0	100	100.0–100.0	100	99.9–100.0
Patella axial	100	100	100.0–100.0	100	100.0–100.0	100	100.0–100.0	100	100.0–100.0
Pelvis AP	100	95	90.7–99.3	99.9	99.8–100.0	96.9	93.5–100.0	99.8	99.7–100.0
Shoulder AP	100	85	78.0–92.0	98.6	98.2–99.0	68	59.8–76.2	99.5	99.2–99.7
Shoulder outlet	100	61	51.4–70.6	99.4	99.1–99.7	78.2	69.0–87.4	98.7	98.2–99.1
Thoracic spine AP	100	97	93.7–100.3	100	100.0–100.0	100	100.0–100.0	99.9	99.8–100.0
Thoracic spine lateral	100	93	88.0–98.0	99.7	99.5–99.9	92.1	86.8–97.3	99.8	99.6–99.9
Wrist AP	100	86	79.2–92.8	99.7	99.5–99.9	90.5	84.6–96.4	99.5	99.3–99.8
Wrist lateral	100	90	84.1–95.9	98.8	98.4–99.2	72.6	64.7–80.4	99.7	99.4–99.9

As for the other performance metrics, the model achieved an average specificity of 99.7%, indicating that, on average, 99.7% of images that were not part of a class were correctly labeled as not belonging to that class. The model achieved an average positive predictive value of 90.8%, indicating that out of all images predicted to belong to a certain class 90.8% of images did actually belong to that class. The average negative predictive value of the model was 99.7%.

### External validation

In the external validation, the overall accuracy of the model in the unseen validation set was 94.0% (95%CI: 93.3–94.6%). The average sensitivity of the model was 93.2%, indicating that 93.2% of images in each category were correctly classified by the model (see Table 3 for performance metrics for each individual class). The sensitivity ranged between 75.0 and 100.0%. Twenty-three out of 30 categories (76.7%) reached a sensitivity of over 90.0%, and 29 out of 30 categories (96.7%) reached a sensitivity of over 80.0%. Only the category finger lateral (75%) scored below 80.0%.

**Table 3:** Performance metrics for the external validation

Category	Number of Images	Sensitivity		Specificity		PPV		NPV	
		Percent	95%CI	Percent	95%CI	Percent	95%CI	Percent	95%CI
Abdomen AP	218	92.2	88.6–95.8	99.7	99.5–99.8	92.2	88.6–95.8	99.7	99.5–99.8
Abdomen left lateral decubitus	29	86.2	73.7–98.8	99.8	99.7–100.0	75.8	61.1–90.4	99.9	99.9–100.0
Ankle AP	75	88	80.6–95.4	99.8	99.7–100.0	89.2	82.1–96.3	99.8	99.7–99.9
Ankle lateral	94	85.1	77.9–92.3	99.8	99.7–99.9	89.9	83.6–96.2	99.7	99.6–99.9
Cervical spine AP	100	99	97.0–100.0	99.7	99.6–99.9	87.6	81.5–93.7	100	99.9–100.0
Cervical spine lateral	150	99.3	98.0–100.0	99.8	99.6–99.9	92.5	88.5–96.6	100	99.9–100.0
Chest lateral	981	99.1	98.5–99.7	99.4	99.2–99.7	97.6	96.6–98.5	99.8	99.7–99.9
Chest PA/AP	1114	89.8	88.0–91.5	100	99.9–100.0	99.9	99.7–100.1	97.4	96.9–97.8
Elbow AP	135	88.9	83.6–94.2	99.9	99.8–100.0	95.2	91.5–99.0	99.7	99.6–99.9
Elbow lateral	123	93.5	89.1–97.9	99.8	99.7–99.9	91.3	86.3–96.2	99.8	99.7–100.0
Finger AP	30	86.7	74.5–98.8	99.6	99.5–99.8	57.8	43.3–72.2	99.9	99.8–100.0
Finger lateral	28	75	59.0–91.0	99.7	99.6–99.8	56.8	40.8–72.7	99.9	99.8–100.0
Foot AP	126	90.5	85.4–95.6	99.8	99.7–99.9	91.9	87.1–96.7	99.8	99.6–99.9
Foot oblique	106	94.3	89.9–98.7	99.7	99.6–99.9	87.7	81.7–93.7	99.9	99.8–100.0
Hand AP	220	97.7	95.8–99.7	100	99.9–100.0	99.5	98.6–100.4	99.9	99.8–100.0
Hand oblique	195	98.5	96.7–100.0	99.9	99.8–100.0	98	96.0–99.9	99.9	99.9–100.0
Hip joint oblique lateral	105	98.1	95.5–100.0	99.9	99.9–100.0	97.2	94.0–100.3	100	99.9–100.0
Knee AP	142	95.8	92.5–99.1	99.7	99.6–99.9	91.3	86.7–95.8	99.9	99.8–100.0
Knee lateral	118	94.1	89.8–98.3	99.8	99.7–99.9	91.7	86.8–96.6	99.9	99.8–100.0
Lumbar spine AP	166	91	86.6–95.3	99.9	99.8–100.0	97.4	94.9–99.9	99.7	99.6–99.9
Lumbar spine lateral	233	91.8	88.3–95.4	99.9	99.8–100.0	96.8	94.5–99.1	99.6	99.5–99.8
Panoramic Radiograph	4	100	100.0–100.0	100	100.0–100.0	100	100.0–100.0	100	100.0–100.0
Patella axial	10	100	100.0–100.0	99.9	99.8–100.0	58.8	35.4–82.2	100	100.0–100.0
Pelvis AP	113	96.5	93.1–99.9	99.9	99.8–100.0	94.8	90.7–98.8	99.9	99.8–100.0
Shoulder AP	166	97	94.4–99.6	99.5	99.3–99.7	86.1	81.1–91.1	99.9	99.8–100.0
Shoulder outlet	117	90.6	85.3–95.9	99.9	99.8–100.0	93.8	89.4–98.2	99.8	99.7–99.9
Thoracic spine AP	87	94.3	89.4–99.1	99.8	99.7–99.9	90.1	84.0–96.2	99.9	99.8–100.0
Thoracic spine lateral	100	98	95.3–100.0	99.5	99.3–99.7	79.7	72.6–86.8	100	99.9–100.0
Wrist AP	116	94	89.6–98.3	99.8	99.7–100.0	93.2	88.6–97.7	99.9	99.8–100.0
Wrist lateral	123	91.9	87.0–96.7	99.6	99.4–99.7	83.1	76.8–89.4	99.8	99.7–99.9

As for the other performance metrics, the model achieved an average specificity of 99.8%, indicating that, on average, 99.8% of images that were not part of a class were correctly labeled as not belonging to that class. The model achieved an average positive predictive value of 88.6%, indicating that out of all images predicted to belong to a certain class 88.6% of images did actually belong to that class. The average negative predictive value of the model was 99.8%.

## Discussion

**Fig. 1:**
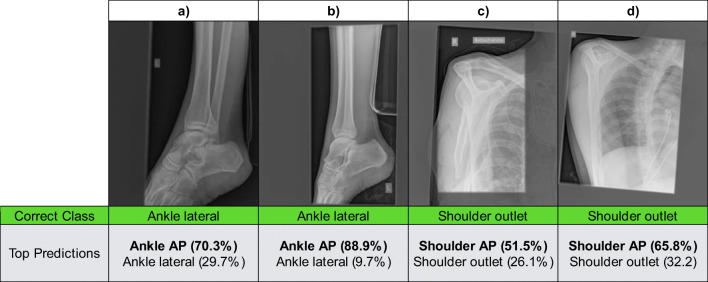
Examples of four images that were misclassified by the neural network. Images **a** and **b** actually belong to the class ankle lateral but were misclassified as ankle AP by the model. Images **c** and **d** actually belong to the class shoulder outlet but were misclassified as shoulder AP. The corresponding prediction values reflect the probability that the image belongs to a certain class, ranging from 0 to 100%. Higher values reflect a higher probability that an image belongs to a certain class

The goal of the present study was to create a neural network for practical applications in the imaging pipeline, e.g., to detect and correct errors in DICOM metadata, to rout radiographs to more specialized networks for abnormality detection, to check that exams are not repeated unnecessarily, to control that the acquired image is the same as the one that was ordered, and to streamline hanging protocols for optimal reporting on images. Our trained model was able to correctly classify the most common types of plain radiographs (e.g., thorax pa, abdomen lateral) and showed good generalizability in the internal (average accuracy: 90.3%) and external validation (average accuracy: 94.0%). However, an overall high accuracy does not necessarily mean that a model will be useful under real-world conditions. One important factor is a comparable level of high performance across all different categories. Combining the results from the internal and external validation set, performance across categories was generally balanced, with only four categories, ankle lateral (79.0%), lumbar spine lateral (77.0%), finger lateral (75.0%), and shoulder outlet (sensitivity: 61.0%) scoring below 80.0%. Taking a closer look at the errors in these categories revealed that the model tended to suggest similar categories and that the correct classification was in many cases the model’s second prediction (see Fig. 1). This may in part be due to suboptimal positioning in some images, for example, where the patient’s pain may have limited the radiographer’s ability to achieve perfect positioning. In contrast, highly standardized and unambiguous image categories (e.g., abdomen left lateral decubitus, patella axial, and chest pa/ap) showed perfect classification results with accuracies of up to 100.0%.

To further assess the performance of our model, it is important to compare its performance with other approaches to classify plain radiographs. Using a CNN and Radon transformation, Khatami et al achieved an accuracy of 90.3% for the validation set of the ImageCLEF2009 medical annotation task. This compares favorably with our own accuracy of 90.3% in the internal validation. However, it is difficult to compare performance on different datasets. To allow further assessment of our model, we will make it available so that other institutions are able to test the performance of the model using their own data.

Our study has some limitations: First, even though the 30 categories included in our study accounted for 81.7% (58,219/71274) of all radiographs performed in 1 year, an ideal system should also include the remaining 72 categories.

Second, the overall accuracy was only 90.3% so that every 1 in 10 images would still need some form of human intervention to be correctly classified. There are several reasons for this: (a) Current approaches are relatively “data-hungry,” which means they need large amounts of images to achieve a high accuracy. Until new techniques emerge that can produce better results with less data, the only option is for multiple institutions to pool their data for less frequent categories to achieve better performance for rare categories. (b) Performance was generally worse for suboptimal images. As mentioned before, performance of the network will depend on the number of low-quality images in the dataset, as high-quality images with little variation are classified more accurately. Because we did test the network on randomly sampled images from our real PACS, the accuracy of our model may be a more accurate predictor of real-world performance than testing the model on a curated data set with only few low-quality images.

With regard to the accuracy achieved in our study, it is important to note, however, that the errors of the model were not random as the model was particularly prone to mistaking similar categories and the correct option was usually among the top suggestions of the model. Furthermore, it would be feasible to use the probability values generated by the model to flag potentially incorrect predictions because we did find that the probability values for incorrect predictions were significantly lower (*M* = 68.2%, *SD* = 21.0%) compared with the probability values for the correct predictions (*M* = 95.2%, *SD* = 10.9%) (*t*(2708) = 35.7, *p* < .001, *d* = 1.61).

Taking into consideration the limitations of our model, the following applications for our AI algorithm are feasible: First, the model can be used to classify images and add or correct DICOM metadata. Even though human review is still needed, the workload can be significantly reduced. Considering that very common categories, such as chest pa/ap or chest lateral, were classified with a relatively high accuracy, large parts of a PACS can be corrected with little error. For instance, in our sample, chest imaging accounted for around 30.4% of radiographs performed in 1 year (21,697/71274). With the categories chest pa/ap and chest lateral achieving an accuracy of 100.0% in the internal validation, 30.4% of images in our sample could have been easily labeled using the AI algorithm. Furthermore, being a relatively low-stakes task compared with the detection of abnormalities, it would be relatively safe to deploy the model.

Second, as part of an automated imaging pipeline, the model can be used to route images to more specialized networks for abnormality detection. For instance, the model can first identify a chest image so that it can then be analyzed by a network specialized for detecting anomalies in chest radiographs [9], abdominal radiographs [10], or musculoskeletal radiographs [11–14]. Again, our model did not achieve perfect accuracy for all classes. However, we think that this does not rule out the deployment of the model. One possible solution for this problem would be to use both the average accuracy of a category as well as individual prediction values to decide how to process images. If an image is from a category with high accuracy (e.g., chest pa/ap) and the prediction value for that particular image is high (> 90.0%), it could be sent straight to a secondary network for abnormality detection. If an image is from a category with low accuracy (e.g., shoulder outlet) and the prediction value for that particular image is also low (< 70.0%), it could be flagged for human review.

In summary, we show that it is possible for a single institution to train a neural network to classify the most common categories of plain radiographs, which can then be used to clean up DICOM metadata or as part of an automated imaging pipeline. To encourage independent review and validation as well as to promote the introduction of new tools that may help radiologists and technicians with routine tasks, the final model will be made publicly available on GitHub (https://github.com/healthcAIr/NNCPR).

## Abbreviations

AI: Artificial intelligence
DICOM: Digital Imaging and Communications in Medicine
JPEG: Joint Photographic Expert Group
PACS: Picture archiving and communication system
